# Three UDP-xylose transporters participate in xylan biosynthesis by conveying cytosolic UDP-xylose into the Golgi lumen in Arabidopsis

**DOI:** 10.1093/jxb/erx448

**Published:** 2017-12-28

**Authors:** Xianhai Zhao, Nian Liu, Na Shang, Wei Zeng, Berit Ebert, Carsten Rautengarten, Qing-Yin Zeng, Huiling Li, Xiaoyang Chen, Cherie Beahan, Antony Bacic, Joshua L Heazlewood, Ai-Min Wu

**Affiliations:** 1 State Key Laboratory for Conservation and Utilization of Subtropical Agro-Bioresources, South China Agricultural University, Guangzhou, China; 2 Guangdong Key Laboratory for Innovative Development and Utilization of Forest Plant Germplasm, College of Forestry and Landscape Architecture, South China Agricultural University, Guangzhou, China; 3 ARC Centre of Excellence in Plant Cell Walls, School of BioSciences, University of Melbourne, Parkville, VIC, Australia; 4 School of BioSciences, University of Melbourne, Parkville, VIC, Australia; 5 State Key Laboratory of Tree Genetics and Breeding, Chinese Academy of Forestry, Beijing, China

**Keywords:** Arabidopsis, cell wall, nucleotide sugar, UDP-xylose, UDP-xylose synthase, UDP-xylose transporter, xylan

## Abstract

UDP-xylose (UDP-Xyl) is synthesized by UDP-glucuronic acid decarboxylases, also termed UDP-Xyl synthases (UXSs). The Arabidopsis genome encodes six UXSs, which fall into two groups based upon their subcellular location: the Golgi lumen and the cytosol. The latter group appears to play an important role in xylan biosynthesis. Cytosolic UDP-Xyl is transported into the Golgi lumen by three UDP-Xyl transporters (UXT1, 2, and 3). However, while single mutants affected in the UDP-Xyl transporter 1 (UXT1) showed a substantial reduction in cell wall xylose content, a double mutant affected in UXT2 and UXT3 had no obvious effect on cell wall xylose deposition. This prompted us to further investigate redundancy among the members of the UXT family. Multiple *uxt* mutants were generated, including a triple mutant, which exhibited collapsed vessels and reduced cell wall thickness in interfascicular fiber cells. Monosaccharide composition, molecular weight, nuclear magnetic resonance, and immunolabeling studies demonstrated that both xylan biosynthesis (content) and fine structure were significantly affected in the *uxt* triple mutant, leading to phenotypes resembling those of the *irx* mutants. Pollination was also impaired in the *uxt* triple mutant, likely due to reduced filament growth and anther dehiscence caused by alterations in the composition of the cell walls. Moreover, analysis of the nucleotide sugar composition of the *uxt* mutants indicated that nucleotide sugar interconversion is influenced by the cytosolic UDP-Xyl pool within the cell. Taken together, our results underpin the physiological roles of the UXT family in xylan biosynthesis and provide novel insights into the nucleotide sugar metabolism and trafficking in plants.

## Teaser

The cytosolic pool of UDP-xylose and its transport into the Golgi lumen play an essential role in xylan and xyloglucan biosynthesis. The equilibrium of these nucleotide sugar pools is controlled by UDP-xylose transporters.

## Introduction

Xylose (Xyl), a major constituent of plant cell walls, is found in both hemicelluloses and pectins. The integral role of Xyl as part of essential structural polymers, such as xylans and xyloglucans (XyGs), exemplifies the significance of this sugar. Xylan is the major hemicellulose present in commelinid monocot (e.g. grasses) (primary and secondary) and non-commelinid monocot and dicot (secondary) cell walls. The backbone of xylan is a linear chain of β-1,4-xylosyl residues that can be decorated with *O*-acetyl (*O*Ac), glucuronic acid (GlcA), 4-*O*-methylglucuronic acid (MeGlcA), and arabinose (Ara) residues (Rennie and Scheller, 2014). As for most polysaccharides, the biosynthesis of the xylan backbone is catalyzed by glycosyltransferases (GTs) that transfer sugars from nucleotide sugar donors on to the growing xylan chain (Peña *et al.*, 2007). Several GTs (IRX9, IRX10, and IRX14, as well as their homologs) (Brown *et al.*, 2007; Lee *et al.*, 2007; Wu *et al.*, 2009; Wu *et al.*, 2010; Lee *et al.*, 2012; Ren *et al.*, 2014) have been implicated in the biosynthesis of the xylan backbone, and there is evidence that these GTs likely function in complexes (Zeng *et al.*, 2010; Ren *et al.*, 2014). Recently, biochemical evidence has demonstrated that these GTs indeed participate in xylan backbone extension (Zeng *et al.*, 2010; Jensen *et al.*, 2014; Urbanowicz *et al.*, 2014). The side-chain decorations on the xylan backbone can vary between plant species and tissues (Scheller and Ulvskov, 2010). In woody tissues of dicots, the GlcA, MeGlcA, and *O*Ac groups are common decorations of the xylan backbone (Zhang *et al.*, 2017), while in grasses Ara substitutions are common (Faik, 2010). The glucuronosyltransferases (GUXs) from the CAZy GT8 family and arabinosyltransferases (XATs) from the GT61 family are responsible for the addition of GlcA and Ara, respectively, on to the xylan backbone (Mortimer *et al.*, 2010; Oikawa *et al.*, 2010; Anders *et al.*, 2012; Lee *et al.*, 2012; Rennie *et al.*, 2012). In contrast to xylan, XyG is the principal hemicellulose in primary walls of dicots, and Xyl is an important constituent of this structure (Scheller and Ulvskov, 2010). The backbone of XyG is a linear chain of β-1,4-glucosyl residues, which in the wall of dicots typically has Xyl, Xyl-galactose (Gal) disaccharide, and Xyl-Gal-fucose (Fuc) trisaccharide side-chain decorations, and the galactosyl residue can be *O*-acetylated (Pauly and Keegstra, 2016). Current biochemical evidence suggests that several genes belonging to the GT34 family encode xylosyltransferases (XylTs) responsible for the addition of xylosyl residues on to the XyG backbone in Arabidopsis (Faik *et al.*, 2002; Cavalier and Keegstra, 2006; Vuttipongchaikij *et al.*, 2012). The subsequent addition of galactosyl residues on to the xylosyl residues requires the galactosyltransferases MUR3 and XLT2, which both belong to the GT47 family (Madson *et al.*, 2003; Jensen *et al.*, 2012). Substitution with a fucosyl residue is facilitated by FUT1/MUR2, a member of the GT37 family (Perrin *et al.*, 1999; Vanzin *et al.*, 2002), while the transfer of *O*Ac on to the XyG polysaccharide is carried out by acetyltransferases belonging to the trichome birefringence-like (TBL) family (Gille *et al.*, 2011; Xiong *et al.*, 2013; Urbanowicz *et al.*, 2014).

Most wall polysaccharides are synthesized in the Golgi lumen, with the exception of cellulose, mixed-linkage glucan, and callose, which are assembled at the plasma membrane. However, the nucleotide sugar donor substrates for the biosynthesis of wall polysaccharides are predominantly generated in the cytosol and need to be transported into the Golgi by nucleotide sugar transporters (NSTs) that are members of the NST/triose phosphate translocator (TPT) superfamily (Knappe *et al.*, 2003). The application of a novel highly selective transporter assay coupled to tandem mass spectrometry has recently led to the identification of numerous new plant NSTs, including UDP-rhamnose/UDP-Gal transporters, UDP-Xyl transporters, UDP-Ara*f* transporters, a GDP-Fuc transporter, and a UDP-uronic acid transporter (Rautengarten *et al.*, 2014; Ebert *et al.*, 2015; Rautengarten *et al.*, 2016; Rautengarten *et al.*, 2017; Saez-Aguayo *et al.*, 2017).

The Golgi-localized XylTs use UDP-Xyl as substrate, which is synthesized in both the cytosol and Golgi lumen by UXSs (Bar-Peled and O’Neill, 2011). The delivery of cytosol-derived UDP-Xyl into the Golgi lumen is facilitated by the three members of the UDP-Xyl transporter family (UXTs) (Ebert *et al.*, 2015). In a recent study it was demonstrated that cytosolic UDP-Xyl represents the major pool used for the biosynthesis of xylan (Kuang *et al.*, 2016), since the *uxs3uxs5uxs6* triple mutant, which lacks the capacity to synthesize UXS-mediated cytosolic UDP-Xyl, displayed an irregular xylem phenotype. However, while single mutants affecting the UDP-Xyl transporter 1 (*UXT1*) gene showed a substantial reduction in wall Xyl content, a double mutant affecting *UXT2* and *UXT3* had no obvious effect on wall Xyl deposition. This phenotype raised questions about functional redundancy between the three *UXT* genes and whether the translocation of UDP-Xyl from the cytosol into the Golgi lumen is required for plant development. Thus, to elucidate the specific UXT functions and to further explore the role of UDP-Xyl partitioning, a *uxt1uxt2uxt3* triple mutant was generated and characterized. Our results highlight the key role that UXTs play in xylan biosynthesis by facilitating the flow of UDP-Xyl from the cytosol into the Golgi lumen. In addition, the *in vivo* UDP-Xyl content appears to have a critical role in balancing nucleotide sugar pools within the plant cell.

## Materials and methods

### Sequence retrieval and phylogenetic analysis

The UXT sequences were obtained from Phytozome (https://phytozome.jgi.doe.gov/). The exon and intron structures of the *UXT* genes were analyzed using Gene Structure Display Server (GSDS 2.0; http://gsds.cbi.pku.edu.cn/) (Hu *et al.*, 2015). Prediction of the transmembrane domains of UXT proteins was conducted using TMHMM v.2.0 (Krogh *et al.*, 2001). Sequences were aligned using the Clustal MUSCLE program (Edgar, 2004) with default parameters. Phylogenetic trees were generated using the neighbor-joining method and bootstrap values generated from 1000 replicates. The respective information on the UXTs is listed in Supplementary Tables S1 and S2 at *JXB* online.

### Plant materials and mutant identification

The T-DNA insertion lines *uxt1* (SALK_086773), *uxt2* (SALK_091753), and *uxt3* (SALK_079036.37.50.x), were obtained from the Arabidopsis Biological Resource Center (ABRC; https://abrc.osu.edu/). Multiple mutants were generated by genetic crosses. Homozygous T-DNA insertion mutants were identified by PCR using genomic DNA as template. Then, RT-PCR analysis was performed to confirm whether the transcripts were either absent or down-regulated in the *uxt* T-DNA insertion lines. Primers are listed in Supplementary Table S3.

### RT-PCR

Total RNA was isolated from leaves of *Arabidopsis thaliana* (L.) Heynh. Columbia-0 (Col-0) using Trizol (Thermo Fisher Scientific). After treatment with DNase I, first-strand cDNA was synthesized using PrimeScript™ RT Master Mix (Takara). The *UXT* genes were amplified using the primers listed in Supplementary Table S3 and the *UBQ10* gene was used as a reference.

### Sectioning of stems

The bottom part of the inflorescence stem of 2-month-old plants was harvested. Sections (50 μm) were cut with a VT1000S vibratome (Leica, Germany). After staining with 0.02% Toluidine Blue O for ~1 min, the sections were washed several times with distilled water and observed by light microscopy (BX43F; Olympus, Japan). For phloroglucinol-HCl staining, two parts of 2% (w/v) phloroglucinol in 95% (v/v) ethanol were mixed with one part concentrated HCl and added to sections. After 10 min incubation at room temperature, photographs were taken using light microscopy. For scanning electron microscopy, sections were vacuum-dried on aluminum foil. Sections were subsequently mounted on stubs, sputter-coated with 40 nm of gold/palladium and viewed using a Hitachi S-4800 FESEM. For the xylan immunolocalization studies, sections were washed with phosphate-buffered saline (PBS: 1.8 mM KH_2_PO_4_, 10 mM Na_2_HPO_4_, 137 mM NaCl, 2.7 mM KCl, pH 7.4) several times and incubated with 5% (w/v) skimmed milk powder for 2 h. The LM10 primary antibody was added and incubated for 1 h; after that, the sections were washed and incubated with the secondary antibody (fluorescein isothiocyanate-conjugated goat anti-rat; Abcam) for 1 h. After final washing steps with PBS (pH 7.4), imaging was conducted using a Zeiss LSM 710 confocal microscope.

### Preparation of alcohol-insoluble residue and cell wall monosaccharide analysis

Alcohol-insoluble residue (AIR) was prepared from stem parts and hydrolyzed in 2 N trifluoroacetic acid for 1 h at 120 °C (Rautengarten *et al.*, 2016). Monosaccharides were separated and quantified by high-performance anion exchange chromatography coupled with pulsed amperometric detection on an ICS 3000 instrument (Thermo Fisher Scientific) using a CarboPac PA20 anion exchange column (Thermo Fisher Scientific) as described previously (Rautengarten *et al.*, 2016).

### ^1^H-NMR spectroscopy of xylo-oligomers

AIR was extracted with 4 M KOH containing 1% (w/v) NaBH_4_. After neutralizing the extracts with glacial acetic acid and dialysis against water, the extracts were lyophilized. Xylan (2 mg) was digested with endo-β-xylanase (Sigma-Aldrich) to release xylo-oligomers. After lyophilization, the xylo-oligomers were dissolved in D_2_O. ^1^H-NMR spectra were recorded with a Bruker AV600 NMR instrument (Zhong *et al.*, 2005). The proton positions and residue identities in NMR spectra were assigned according to Zhong *et al.* (2017).

### Size-exclusion chromatography

The weight-average (Mw) and number-average (Mn) molecular weights of the hemicellulose fractions were determined by size-exclusion chromatography (SEC) on a PL aquagel-OH 50 column (300 × 7.7 mm; Polymer Laboratories Ltd). Elution was conducted at 30 °C with 0.02 N NaCl in 5 mM sodium phosphate buffer (pH 7.5) at 0.5 ml min^–1^. Calibration was performed using pullulan standards (Mw of 738, 12200, 100000, 1600000; Polymer Laboratories Ltd).

### Saccharification

AIR (2 mg) was pretreated with water by autoclaving for 30 min at 120 °C. After washing with water several times, cellulase (Sigma-Aldrich) and endo-β-xylanase (Sigma-Aldrich) were added separately. The samples were incubated at 37 °C for 24 h under constant shaking at 800 rpm. The sugar content of enzymatic hydrolysates was quantified using the phenol-sulfuric assay (Zhao *et al.*, 2017).

### Nucleotide sugar extraction and detection

The shoots of 21-day-old seedlings grown on Murashige and Skoog plates were collected. The extraction and detection of nucleotide sugars was performed according to Rautengarten *et al.* (2014). LC-MS/MS analysis was performed using porous graphitic carbon as the stationary phase on a 1100 series HPLC system (Agilent Technologies) and a 4000 QTRAP LC-MS/MS system (Sciex) equipped with a TurbolonSpray ion source, as described previously (Rautengarten *et al.*, 2014).

### ELISA analysis

AIR was extracted using 4 M KOH containing 1% (w/v) NaBH_4_. After neutralizing with glacial acetic acid, 50 μl samples of the extracts were added into 96-well plates (Costar 3598) and dried in an incubator at 37 °C overnight. The plates were blocked with 200 μl of 5% (w/v) skimmed milk powder in PBS (pH 7.4) for 1 h. After washing several times, the primary antibodies (LM10, LM11, LM15, and LM25) were applied; the wells were then washed several times and the secondary antibody (peroxidase-conjugated goat anti-rat IgG antibody, Abcam) was applied. The wells were again washed several times and a Pierce TMB substrate kit (Thermo Fisher Scientific) was used for detection. The reaction was stopped by adding 50 μl of 0.5 N sulfuric acid to each well and subsequently the output of each well was read as the difference between A_450_ and A_655_ using an EnSpire plate reader (PerkinElmer) (Pattathil *et al.*, 2010).

## Results and discussion

### Evolution of *UXT* genes in land plants

The Arabidopsis genome encodes three UXT proteins, with UXT2 and UXT3 displaying the highest level of identity at ~92% (Supplementary Table S1). To examine the evolutionary history of the *UXT* gene family in land plants, UXT homologs were obtained from a range of plant species, including *Physcomitrella patens*, *Selaginella moellendorffii*, *Oryza sativa*, *Sorghum bicolor*, *Picea abies*, *Eucalyptus grandis*, and *Populus trichocarpa*. The phylogenetic analysis showed that UXTs in land plants generally fall into two clades (clade I and clade II) (Supplementary Fig. S1). Consistent with the high sequence identity for Arabidopsis UXT2 and UXT3, these two proteins were classified together into clade II with *Physcomitrella*, *Selaginella*, and *Picea*, and UXT1 was classified into clade I (Supplementary Fig. S1). From these results, we inferred that the UXTs diverged early in the evolution of land plants. Further, the phylogenetic analysis indicated that UXT2 and UXT3 are conserved in all plants, with UXT1 being less important in lower plants but conserved in angiosperms to potentially satisfy greater xylan biosynthesis (Supplementary Fig. S1). Interestingly, UXT1 of Arabidopsis is localized to both the endoplasmic reticulum and Golgi, while UXT2 and UXT3 are located to Golgi (Ebert *et al.*, 2015). Furthermore, UXT1 exhibited higher UDP-Xyl transport activity (Ebert *et al.*, 2015), indicating that UXT1 might play important roles in the development of angiosperms.

### Plant growth and secondary wall thickening are affected in *uxt* multiple mutants

**Fig. 1.**
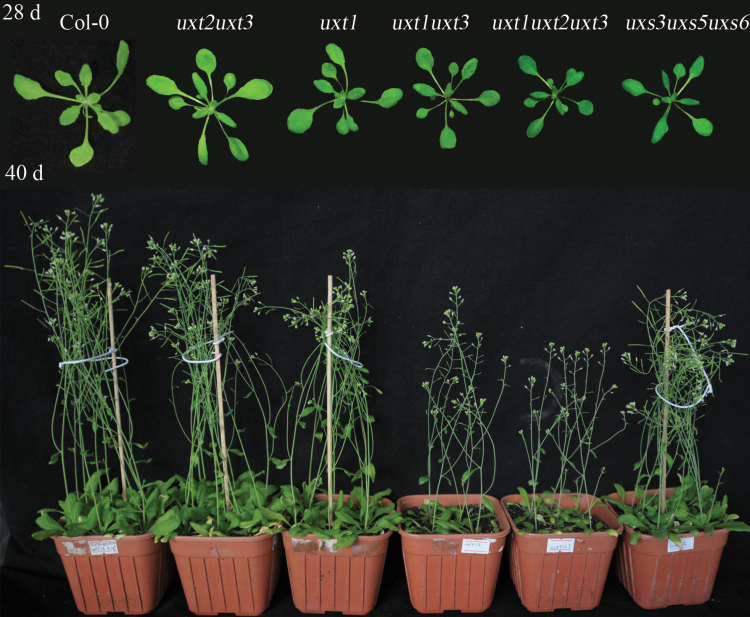
Representative morphological phenotypes of 28-day-old (upper panel) and 40-day-old (lower panel) *uxt* mutant plants in comparison to the wild type (Col-0) and the *uxs3uxs5uxs6* triple mutant.

**Fig. 2.**
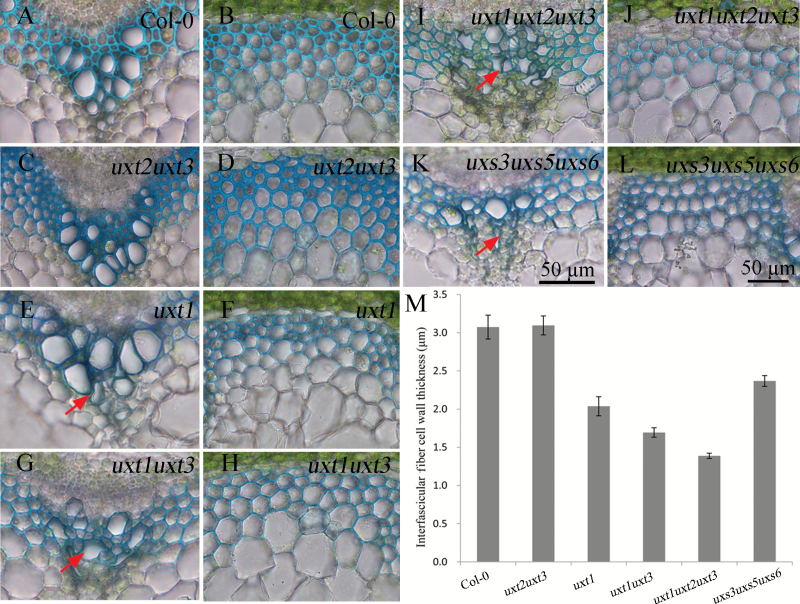
Cross-sections of stem inflorescences of 40-day-old *uxt* and *uxs* mutant plants in comparison to wild type (Col-0). (A, C, E, G, I, K) Xylem vessels. (B, D, F, H, J, L) Fiber cell walls. The arrows highlight xylem vessels that have partially collapsed in (E) *uxt1*, (G) *uxt1uxt3*, (I) *uxt1uxt2uxt3*, and (K) *uxs3uxs5uxs6*. (M) Average thickness of interfascicular fiber cell walls (n=20 cells) from four individual stems for each genotype. Error bars represent SD.

To identify functional roles for the three Arabidopsis UXTs, genetic crosses between UXT mutants were conducted. Owing to the genetic linkage between the *UXT1* and *UXT2* genes, the *uxt1uxt2uxt3* triple mutant was obtained from the *uxt1uxt3* and *uxt2uxt3* double mutants, without generation of an *uxt1uxt2* double mutant. RT-PCR analysis confirmed the identity of the knockout lines (Supplementary Fig. S2). Consistent with the observations by Ebert *et al.*, (2015), *uxt2uxt3* double mutants did not show any morphological changes when compared with the wild type. However, *uxt1uxt3* and *uxt1uxt2uxt3* mutants exhibited delayed growth, a darker leaf color, and shorter stems (Fig. 1 and Supplementary Fig. S3). Transverse sections from stems of these mutants were examined to assess the effects on structures rich in xylan. Consistent with the observed growth phenotypes, the vessels of *uxt1uxt3* and *uxt1uxt2uxt3* mutants were severely collapsed (Fig. 2G, I). In contrast, vessels of *uxt1* mutant plants showed only a mild deformation (Fig. 2E), and *uxt2uxt3* mutant vessels showed no apparent alterations (Fig. 2C). Compared with the *uxt2uxt3* mutant, *uxt1uxt3* had a more severe phenotype, supporting the more prominent role of UXT1 relative to UXT3 in *Arabidopsis*. The *uxt1uxt2uxt3* triple mutant showed the most severe vessel defects and had the thinnest interfascicular fiber cell walls, corresponding to the observed developmental defects of shorter stems (Fig. 2I, J, M). Transmission electron microscopy further confirmed that the interfascicular fiber cell wall thickness of the *uxt1uxt2uxt3* triple mutant was reduced to 20–40% of that of the wild type (Supplementary Fig. S4). The *uxt1uxt2uxt3* mutant phenotype is reminiscent of that of the irregular xylem (*irx*) mutants, which have collapsed vessels and reduced secondary cell walls (Brown *et al.*, 2005). This finding is surprising given the availability of luminal UDP-Xyl, but confirms the importance of the cytosolic UDP-Xyl pool for xylan biosynthesis.

### Xylan content is reduced in *uxt* mutants

**Fig. 3.**
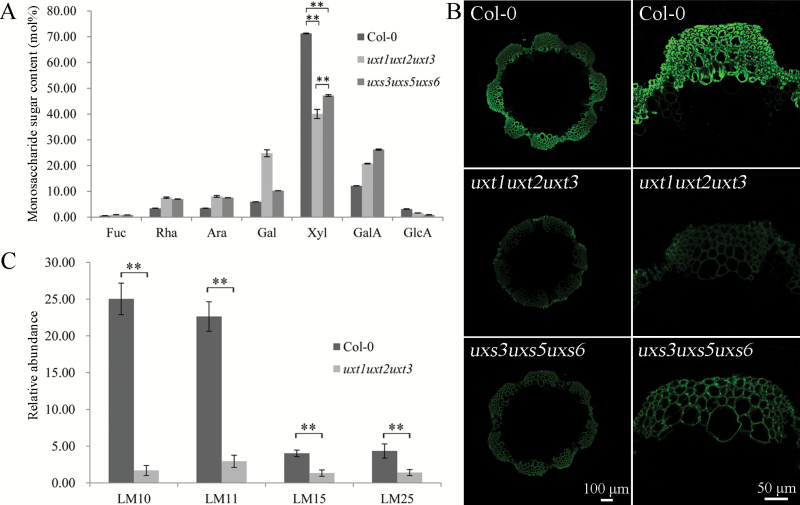
Analysis of cell wall characteristics and composition. (A) Monosaccharide composition of *uxt1uxt2uxt3* and *uxs3uxs5uxs6* mutant stems, determined by high-performance anion exchange chromatography coupled with pulsed amperometric detection. Results are means ±SD (*n*=6). **P<0.01, *t* test. (B) Immunolocalization analysis of LM10 epitopes in stem sections from the *uxt1uxt2uxt3* and *uxs3uxs5uxs6* mutants. (C) ELISA analysis of xylan and XyG from *uxt1uxt2uxt3* cell walls using the LM10, LM11, LM15, and LM25 antibodies. LM10 and LM11 are antibodies specific for xylan epitopes, whereas LM15 and LM25 recognize XyG epitopes. Results are means ±SD (*n*=6), **P<0.01, *t* test.

The impact of eliminating UDP-Xyl transport and cytosolic UDP-Xyl biosynthesis on xylan production was further investigated by analyzing the monosaccharide composition of the basal part of the stem of the triple *uxt1uxt2uxt3* and *uxs3uxs5uxs6* mutants and performing immunolocalization studies on stem sections using the LM10 monoclonal antibody, which recognizes xylan epitopes (McCartney *et al.*, 2005). Both the *uxt1uxt2uxt3* and *uxs3uxs5uxs6* triple mutants showed reduced cell wall Xyl content (Fig. 3A). In accordance with its shorter stem relative to wild type and the other mutants (Fig. 1), the *uxt1uxt2uxt3* mutant showed lower levels of Xyl and less LM10 labeling than the *uxs3uxs5uxs6* mutant and wild type (Fig. 3A, B) (McCartney *et al.*, 2005). Moreover, the ELISA results showed that XyG content in the *uxt1uxt2uxt3* mutant was also reduced relative to wild type (Fig. 3C). The observed difference in phenotypes between the *uxs3uxs5uxs6* and *uxt1uxt2uxt3* triple mutants may be due to the leaky mutation of the *UXS5* gene in the *uxs3uxs5uxs6* mutant (Kuang *et al.*, 2016) and/or might be explained by the existence of UDP-apiose/UDP-Xyl synthases (Mølhøj *et al.*, 2003), which could still provide cytosolic UDP-Xyl in the *uxs3uxs5uxs6* triple mutant. However, since both triple mutants exhibit similar phenotypic trends, the differences may also reflect technical variations in the data.

### The structure of xylan is modified in the *uxt1uxt2uxt3* mutant

**Fig. 4.**
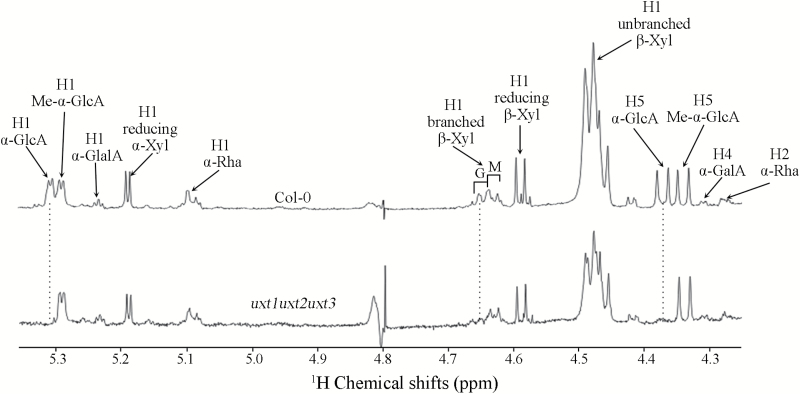
^1^H-NMR spectra of endo-xylanase-digested cell wall material of the *uxt1uxt2uxt3* mutant. G denotes xylosyl residues branched with α-GlcA, and M denotes xylosyl residues branched with Me-α-GlcA. Note the loss of resonance of H1 and H5 of α-GlcA at 5.31 and 4.36 ppm, respectively, as well as H1 of β-Xyl branched with α-GlcA at 4.64 ppm.

Previously, an increase in the proportion of MeGlcA relative to GlcA was observed in xylan-related mutants including the *uxs* triple mutant and the *uxt1* single mutant (Ebert *et al.*, 2015; Kuang *et al.*, 2016; Zhong *et al.*, 2017). Therefore, ^1^H NMR spectroscopy of endo-xylanase-digested AIR (wall material) of *uxt1uxt2uxt3* was undertaken. There were no signals for H1 and H5 of α-GlcA at 5.31 and 4.36 ppm in the *uxt1uxt2uxt3* triple mutant, respectively. H1 of β-Xyl branched with α-GlcA at 4.64 ppm was also absent in the *uxt1uxt2uxt3* triple mutant (Fig. 4). However, the wild type showed both α-GlcA at 5.31 and 4.36 ppm, and Me-α-GlcA at 5.29 and 4.33 ppm, respectively (Fig. 4). These observations indicate that all the α-GlcA residues in the *uxt1uxt2uxt3* mutant were methylated.

In a previous study, we determined that the molecular weight of xylan was reduced in the *uxs3uxs5uxs6* mutant (Kuang *et al.*, 2016). To explore whether this is also true for the *uxt1uxt2uxt3* mutant, 1 M KOH and 4 M KOH soluble extracts were prepared from AIR and analyzed by size-exclusion chromatography. Both the 1 M and 4 M KOH soluble extracts of the *uxt1uxt2uxt3* triple mutant showed a reduced molecular weight (Supplementary Fig. S5). These results imply that the rate of transport of UDP-Xyl into the Golgi is a limiting factor in the elongation of the xylan backbone.

**Fig. 5.**
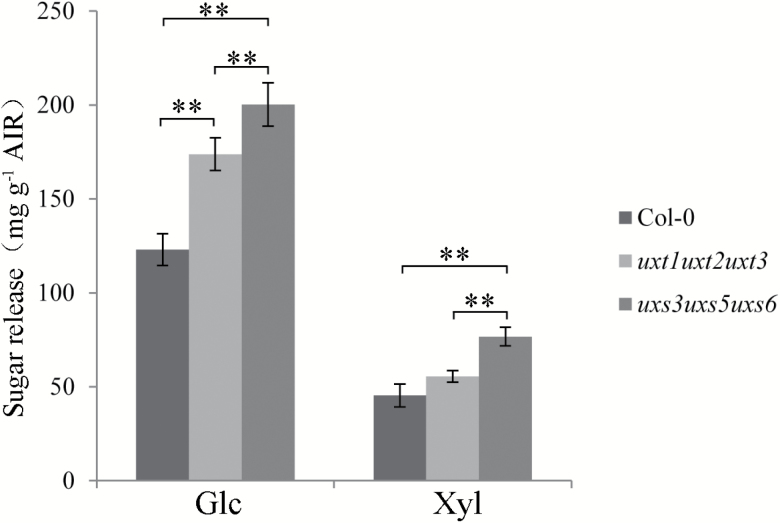
Increased saccharification digestibility of the *uxt1uxt2uxt3* and *uxs3uxs5uxs6* mutants compared with wild type. Results are mean ±SD (*n*=6). **P<0.01, *t* test.

In addition, both the *uxt1uxt2uxt3* and *uxs3uxs5uxs6* mutants exhibited increased enzymatic digestibility compared with the wild type (Fig. 5). However, the saccharification efficiency of cell wall material from the *uxt1uxt2uxt3* mutant was lower than that from the *uxs3uxs5uxs6* mutant (Fig. 5). Taken together, these data led us to conclude that the xylan structure in the *uxt1uxt2uxt3* mutant was modified in comparison to that of the wild type.

### Nucleotide sugar compositions differ in the *uxt* and *uxs* triple mutants

**Fig. 6.**
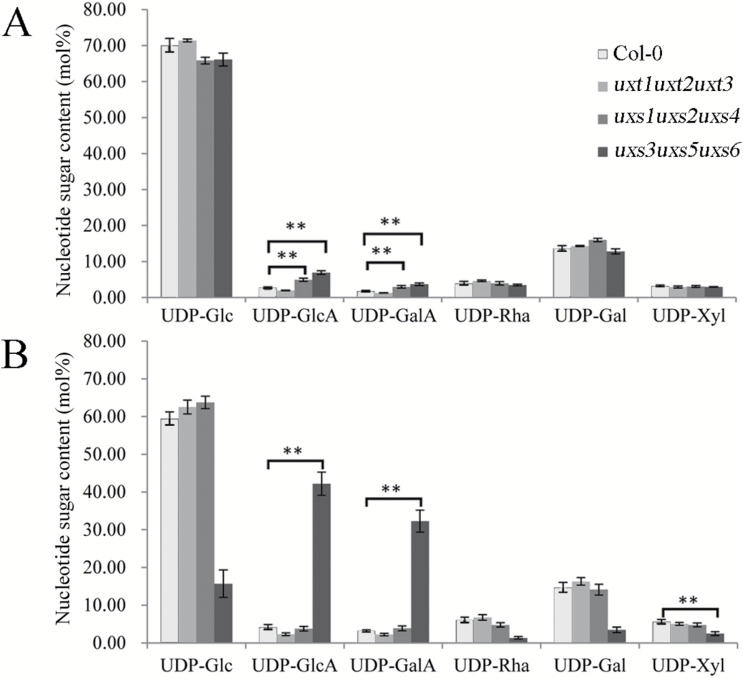
Nucleotide sugar composition of *uxt* and *uxs* mutant plants. Nucleotide sugars extracted from leaves of 21-day-old seedlings grown on Murashige and Skoog plates (A) and 5 cm of the basal stems of 40-day-old plants (B) were analyzed by LC-MS/MS. Results are mean ±SD (*n*=6). **P<0.01, *t* test.

To assess the variation of nucleotide sugar levels in the mutant plants, nucleotide sugar measurements were performed (Rautengarten *et al.*, 2014). For this analysis, leaves of 21-day-old seedlings grown on Murashige and Skoog plates and the 5 cm basal part of stems of 40-day-old plants were selected to represent primary (Somerville *et al.*, 2004) and secondary cell walls (Brown *et al.*, 2005), respectively. Nucleotide sugar levels of the *uxt1uxt2uxt3* mutant were similar to those observed in the wild type in both tissues (Fig. 6A, B). The minor changes in UDP-Xyl levels in the *uxt1uxt2uxt3* mutant compared with wild type were not anticipated, as we expected the levels to be higher in the mutant. This observation supports the existence of a UDP-Xyl feedback mechanism. Previous reports have shown that UDP-Xyl acts as a feedback inhibitor of UXS (Harper and Bar-Peled, 2002), UDP-Glc dehydrogenase (UGD) (Stewart and Copeland, 1999), and UDP-GlcA 4-epimerase (GAE) (Bar-Peled and O’Neill, 2011). Therefore, an accumulation of cytosolic UDP-Xyl in the *uxt1uxt2uxt3* mutant may be inhibiting these enzymes. The *uxs1uxs2uxs4* mutant, which lacks luminal UDP-Xyl biosynthesis, also showed little variation in nucleotide sugar levels, including UDP-Xyl content, compared with the wild type; although the amount of UDP-GlcA and UDP-GalA content was slightly higher in leaf samples (Fig. 6A). By contrast, both leaf and stem samples of the *uxs3uxs5uxs6* mutant showed an accumulation of UDP-GlcA and UDP-GalA, in particular from stem samples (Fig. 6B). In contrast to all other mutants analyzed, only stem samples from the *uxs3uxs5uxs6* triple mutant showed a significant reduction in the proportion of UDP-Xyl content (Fig. 6B). Interestingly, although Xyl is the second most abundant sugar in secondary cell walls and demand for this substrate in xylan-producing cells should be high, no significant increase in the amount of UDP-Xyl in wild-type stem samples compared with wild-type leaf samples was observed. The reduction of cytosolic UDP-Xyl observed in the *uxs3uxs5uxs6* mutant may have minimized inhibitory effects on UGD and GAE and thus led to the observed increase in UDP-GlcA and UDP-GalA, while the decrease in UDP-Glc might be due to feedback from increased UDP-GlcA levels. Together, the findings of the nucleotide sugar analysis of the mutant plants revealed that cytosolic UXSs supply most of the essential UDP-Xyl for the stem of Arabidopsis plants and that UDP-Xyl plays an important role in regulating nucleotide sugar levels in stems.

### Changes in cell wall composition likely reduce the fertility of the *uxt* triple mutant

**Fig. 7.**
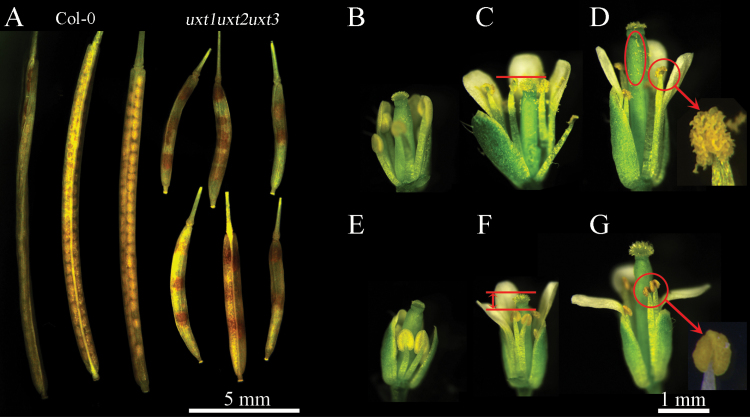
The siliques and flowers of wild-type Col-0 and *uxt1uxt2uxt3* mutants. (A) Siliques of wild-type Col-0 and *uxt1uxt2uxt3* mutants. (B–G) Flowers of wild-type Col-0 (B, C, D) and *uxt1uxt2uxt3* mutants (E, F, G). (B) and (E) Flowers at the pre-pollination stage. (C) and (F) Flowers at the exact-pollination stage. The tops of the stamens are either parallel to or just above the pistil in wild-type Col-0. The highest position of the stamens in the *uxt1uxt2uxt3* mutant is just below the pistil. (D) and (G) Flowers at the post-pollination stage. There is abundant pollen on the style and anther of the wild-type Col-0, whereas only a few pollen grains can be observed on the style of the *uxt1uxt2uxt3* triple mutant. The anther of the *uxt1uxt2uxt3* mutant appears to be indehiscent.

In addition to the observed growth phenotype, *uxt1uxt2uxt3* mutant plants were significantly affected in silique development and seed production (Fig. 7A). To verify whether either the male or female gametophytes of the *uxt1uxt2uxt3* mutant were aborted, styles were pollinated with wild-type and *uxt1uxt2uxt3* mutant pollen. Both crosses resulted in seed production and normal siliques, indicating that both the male and female gametes were unaffected in the *uxt1uxt2uxt3* mutant (Supplementary Fig. S6). Analysis of flowers from the *uxt1uxt2uxt3* mutant indicated that most anthers had short filaments that could not reach the stigma (Fig. 7B–G). This defect is similar to that of the *atxth28* mutant, whose self-pollination capacity is decreased (Kurasawa *et al.*, 2009). The XyG endotransglucosylase/hydrolase (XTH) 28 gene is mutated in the *atxth28* plant and the structure of XyG in this mutant is affected and as a result inhibits stamen filament growth (Kurasawa *et al.*, 2009). The results of ELISA on leaf material showed that, besides xylan, the XyG content was also reduced in the *uxt1uxt2uxt3* triple mutant, thus indicating that the decrease in XyG may be responsible for the aberrant filament growth (Fig. 7E).

**Fig. 8.**
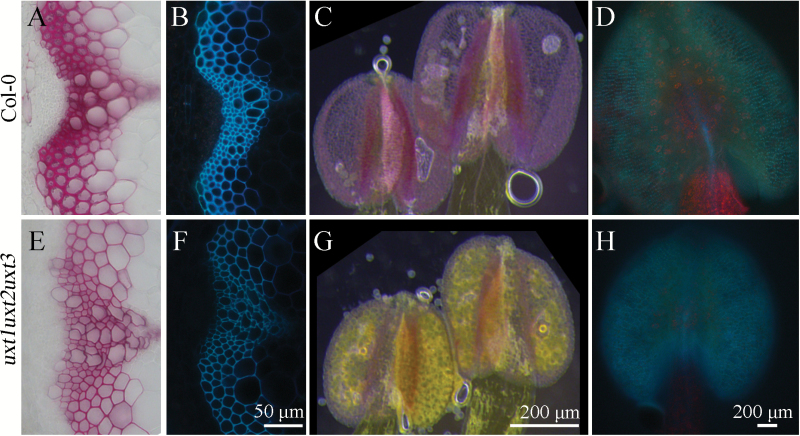
Phloroglucinol-HCl staining (A, C, E, G) and UV fluorescence (B, D, F, H) of stem sections and anthers of the *uxt1uxt2uxt3* mutant and wild-type Col-0. The *uxt1uxt2uxt3* mutant shows reduced lignin content in both stems and anthers.

Furthermore, anthers of the *uxt1uxt2uxt3* mutant were indehiscent and little pollen was observed on the style of the *uxt1uxt2uxt3* mutant (Fig. 7G). This observation is comparable to the xylan mutant, *irx8*, in which anthers are also indehiscent, likely as a result of reduced lignin content (Hao *et al.*, 2014). Similarly, in the *uxt1uxt2uxt3* mutant, phloroglucinol-HCl staining and UV visualization indicated that both the stems and anthers had a reduced lignin content compared with the wild type (Fig. 8). Thus, the reduced seed production observed for *uxt1uxt2uxt3* mutant plants might be a result of the reduced xylan, XyG, and lignin content in the anther cell wall, which consequently leads to inhibition of growth and elongation of the filaments.

**Fig. 9.**
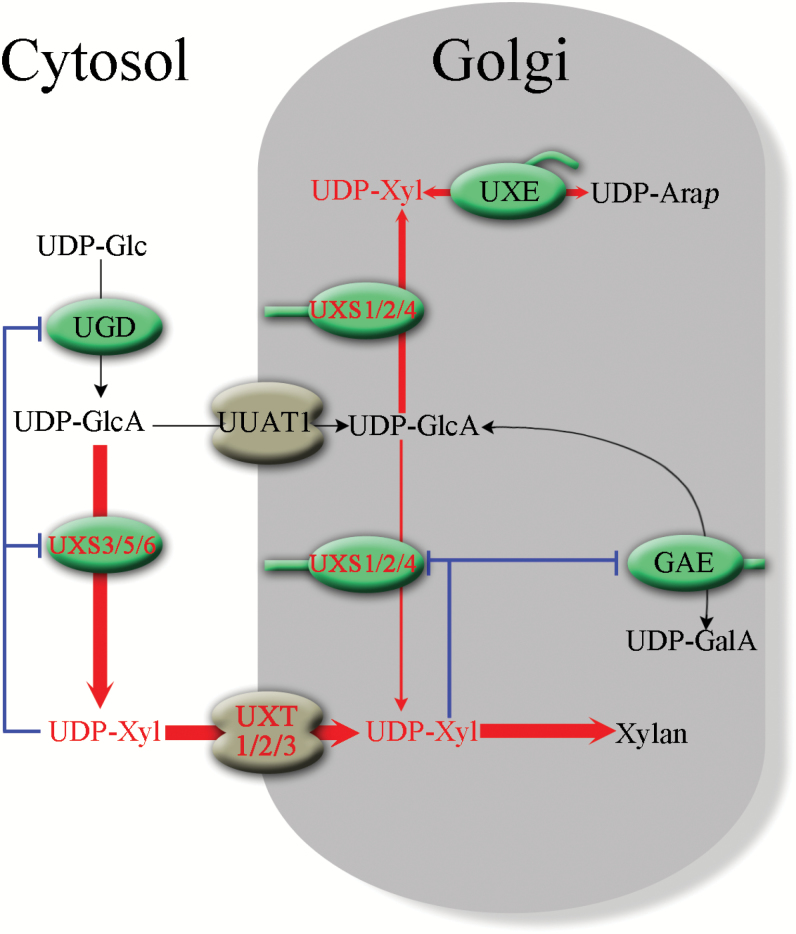
Model outlining the translocation and transformation of UDP-Xyl. GAE, UDP-GlcA 4-epimerase; UGD, UDP-Glc dehydrogenase; UXT, UDP-Xyl transporter; UUAT, UDP-uronic acid transporter; UXE, UDP-Xyl 4-epimerase. Blue lines indicate the feedback inhibition of UDP-Xyl on UXS, UGD, and GAE enzymes. Red lines indicate the flow of UDP-Xyl. The thickness of the red lines indicates the relative amount of nucleotide sugar allocated to different biosynthetic functions.

In summary, this study highlights the key role that UXTs play in xylan biosynthesis by regulating the flow of UDP-Xyl from the cytosol to the Golgi lumen, as depicted schematically in Fig. 9. The cytosolic pool of UDP-Xyl is proposed to be directly utilized by XylTs for xylosylation reactions, whereas the majority of luminal UDP-Xyl synthesized by Golgi-localized UXSs is converted to UDP-Ara*p* (Rautengarten *et al.*, 2017). This selectivity for UDP-Xyl within the Golgi lumen after transport would likely occur through the formation of protein complexes between UXTs and XylTs. Channeling of UDP-Xyl into glucuronoxylan has been observed in Arabidopsis specifically overexpressing UXT1, and this points to an association between UXT1 and specific XylTs (Ebert *et al.*, 2015). Further support for this model is provided by the observations that the *uxt* triple mutant (i) contained less Xyl and more Ara in the cell wall (Fig. 3A) and (ii) showed a dramatic reduction in cell wall Xyl deposition and a severely affected growth phenotype without functional UXT that could transport cytosolic UDP-Xyl into the Golgi lumen. In addition, the *in vivo* UDP-Xyl content seems to have a critical role in balancing the nucleotide sugar pools within the plant cell.

## Supplementary data

Supplementary data are available at *JXB* online.

Fig. S1. Phylogenetic tree of UXTs from land plants.

Fig. S2. T-DNA insertion lines used in this study.

Fig. S3. The plant heights (A) and leaf number (B) of 50-day-old wild-type and *uxt1uxt2uxt3* mutant plants.

Fig. S4. Scanning electron microscopy of cross-sections from the *uxt1uxt2uxt3* mutants showing reduced thickness of fiber cells.

Fig S5. Molecular weight distribution of xylan from wild-type and *uxt1uxt2uxt3* mutant plants.

Fig. S6. The *uxt1uxt2uxt3* mutant siliques with and without artificial pollination.

Table S1. Coding region nucleotide (upper portion of matrix) and amino acid (bottom portion of matrix) sequence pairwise comparisons (% similarity) between Arabidopsis UXTs.

Table S2. UXTs used for gene structure analysis and phylogenetic tree construction.

Table S3. List of primers used in this study.

Supplementary Files
