# Supplementary material for: Three UDP-xylose transporters participate in xylan biosynthesis by conveying cytosolic UDP-xylose into the Golgi lumen in Arabidopsis
Source: J Exp Bot. 2017 Dec 28;69(5):1125–34. doi: 10.1093/jxb/erx448 (PMC6018967; doi:10.1093/jxb/erx448)
Supplement: Supplementary Files [file erx448_suppl_supplementary_files.pdf]

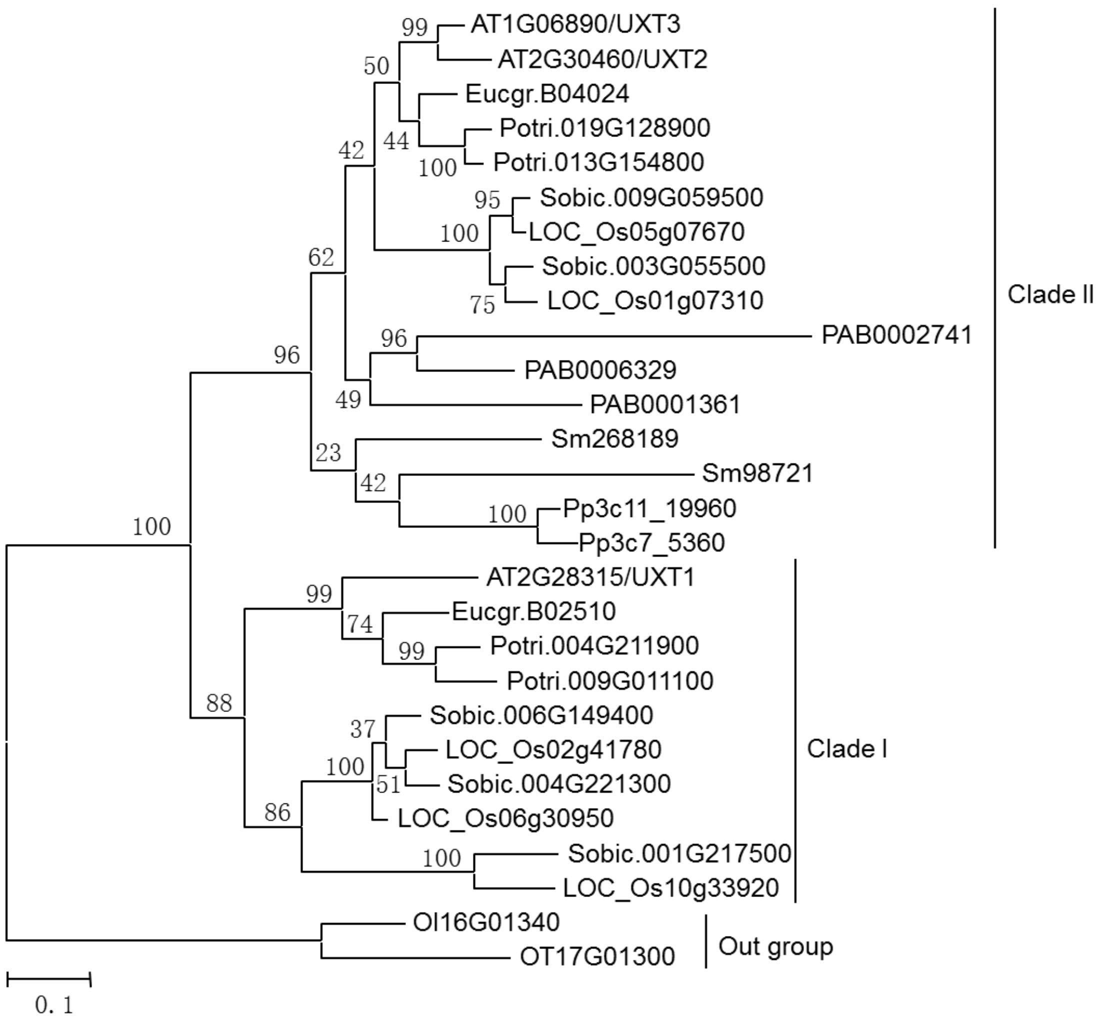

Fig. S1. Phylogenetic tree of UXTs from land plants. They are *Arabidopsis, Physcomitrella patens, Selaginella moellendorffii, Picea abies* (PAB), *Oryza sativa, Sorghum bicolor, Eucalyptus grandis,* and *Populus trichocarpa*. Numbers on branches indicate the bootstrap percentage values calculated from 1,000 replicates. The green alga *Ostreococcus tauri* (OT) and *Ostreococcus lucimarinus* (OL) UXT protein was used as the outgroup. Details of sequences used are outlined in Table S2.

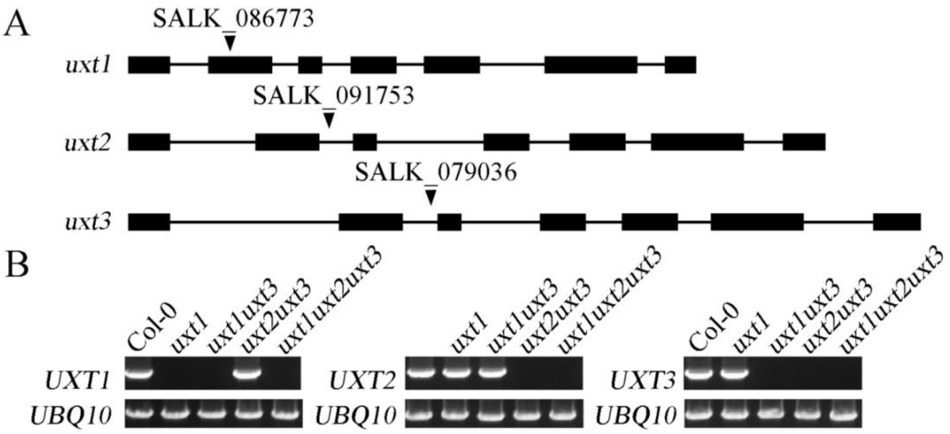

Fig. S2. The T-DNA insertion lines used in this study.

(A) Diagrams of the exons (filled boxes) and introns of the *UXT* genes. T-DNA insertions are indicated by arrows.

(B) RT-PCR analysis confirmed the absence of transcripts in the *uxt* T-DNA insertion lines. PCR amplified *UBQ10* was used as a loading control.

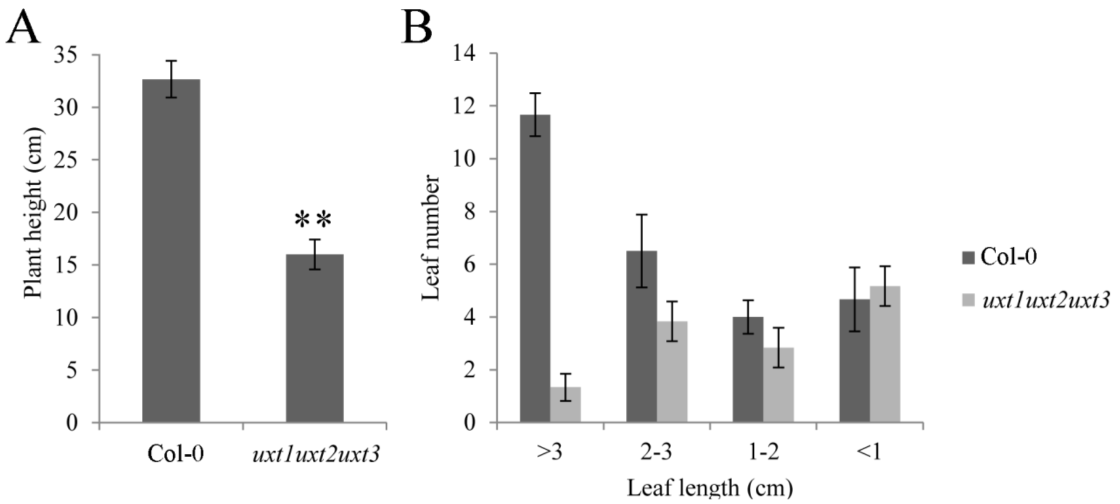

Fig. S3. The plant heights (A) and leaf number (B) of 50-day-old wild type and *uxt1uxt2uxt3* mutant plants. Results are means ± SD (*n*=6, ***P*<0.01, *t* test).

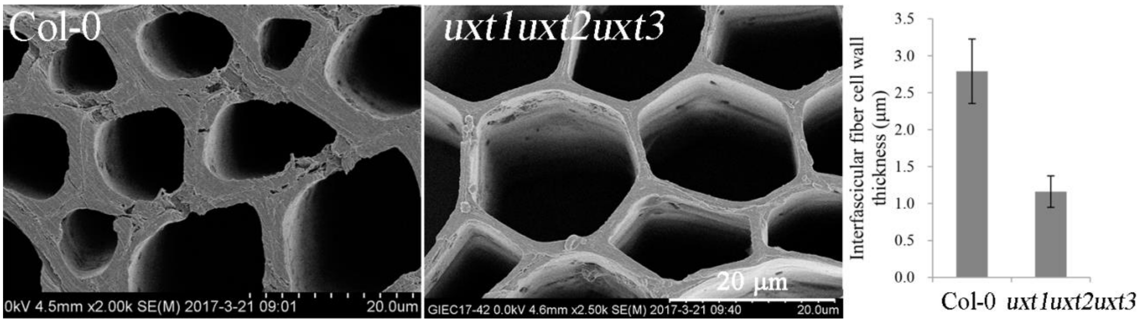

Fig. S4. Scanning electron microscopy on cross sections of the *uxt1uxt2uxt3* mutant showed reduced thickness of fiber cells.

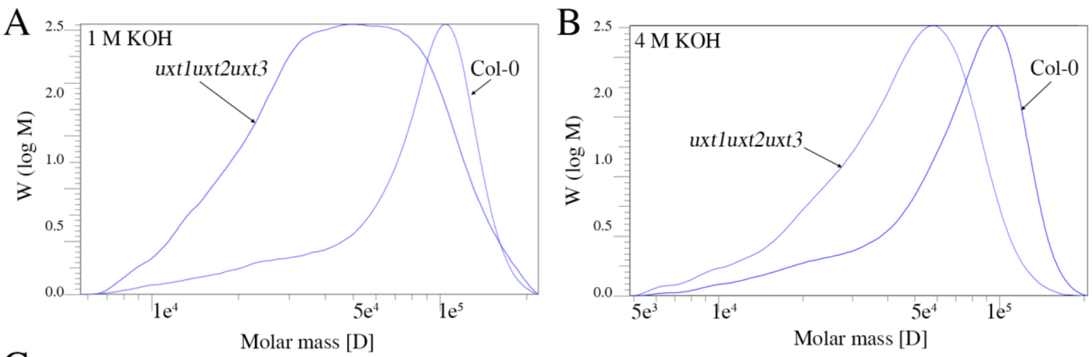

C

	1 M KOH		4 M KOH	
	Col-0	*uxt1uxt2uxt3*	Col-0	*uxt1uxt2uxt3*
Mn :	51667	35207	57078	28009
Mw :	76927	51268	85603	48930
Mw/Mn	1.49	1.46	1.50	1.75

Fig. S5. The molecular weight distribution of xylan of wild type Col-0 and *uxt1uxt2uxt3* mutants. Xylan extracted with 1 M KOH (A) and 4 M KOH (B) from wild type Col-0 and *uxt1uxt2uxt3* mutants were separated by size- exclusion chromatography. (C) The number-average molecular weight Mn and weight-average molecular weight Mw of the xylan extracted with 1 M KOH and 4 M KOH.

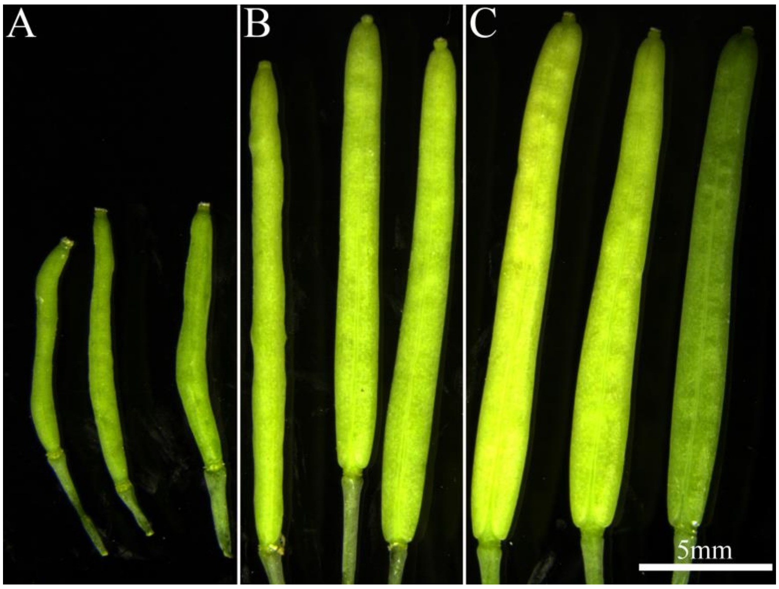

Fig. S6. The *uxt1uxt2uxt3* mutant siliques with and without artificial pollination.

(A) The *uxt1uxt2uxt3* mutant siliques with no artificial pollination.

(B) The *uxt1uxt2uxt3* mutant siliques pollinated with wild type Col-0 pollen.

(C) The *uxt1uxt2uxt3* mutant siliques hand-pollinated with *uxt1uxt2uxt3* mutant pollen.

Table S1. Coding region nucleotide (upper portion of matrix) and amino acid (bottom portion of matrix) sequence pairwise comparisons (% similarity) between *Arabidopsis UXTs*.

	UXT1	UXT2	UXT3
UXT1	-	64.84	66.02
UXT2	65.79	-	87.19
UXT3	66.96	91.78	-

Table S2. The UXTs used in gene structure analysis and phylogenetic tree construction. All of the UXTs were obtained from phytozome (https://phytozome.jgi.doe.gov).

Gene symbol	Species
Potri.019G128900	*Populus trichocarpa*
Potri.013G154800	*Populus trichocarpa*
Eucgr.B04024	*Eucalyptus grandis*
AT2G30460	*Arabidopsis*
AT1G06890	*Arabidopsis*
LOC_Os01g07310	*Oryza sativa*
Sobic.003G055500	*Sorghum bicolor*
LOC_Os05g07670	*Oryza sativa*
Sobic.001G217500	*Sorghum bicolor*
Sm268189	*Selaginella moellendorffii*
Pp3c11_19960	*Physcomitrella patens*
Pp3c7_5360	*Physcomitrella patens*
Sm98721	*Selaginella moellendorffii*
Potri.004G211900	*Populus trichocarpa*
Potri.009G011100	*Populus trichocarpa*
Eucgr.B02510	*Eucalyptus grandis*
AT2G28315	*Arabidopsis*
LOC_Os10g33920	*Oryza sativa*
Sobic.009G059500	*Sorghum bicolor*
Sobic.004G221300	*Sorghum bicolor*
LOC_Os02g41780	*Oryza sativa*
LOC_Os06g30950	*Oryza sativa*
Sobic.006G149400	*Sorghum bicolor*
PAB0006329	*Picea abies*
PAB0002741	*Picea abies*
PAB0001361	*Picea abies*
Ol16G01340	*Ostreococcus lucimarinus*
OT17G01300	*Ostreococcus tauri*

Table S3. Primer list.

Gene	Forward primer	Reverse primer
T-DNA insertion lines		
uxt1	CTTCAGCTCAATTTTGTTGGC	AGAAGAGAAAATGCCCATTGG
uxt2	AGAAGTTGGGTGGATGAAACC	TGGTTTGATTCCACAGGAAAC
uxt3	GTTCTGATTGGTTAGGAGCCC	CGAGAATTGCCTTCATGATTG
RT-PCR		
uxt1	CTTCAGCTCAATTTTGTTGGC	AGAAGAGAAAATGCCCATTGG
uxt2	AGAAGTTGGGTGGATGAAACC	TGGTTTGATTCCACAGGAAAC
uxt3	GTTCTGATTGGTTAGGAGCCC	CGAGAATTGCCTTCATGATTG
UBQ10	GGCCTTGTATAATCCCTGATGAATAAG	AAAGAGATAACAGGAACGGAAACATAGT
